# Pancreatic Sarcoidosis: A Rare Manifestation of Systemic Sarcoidosis

**DOI:** 10.7759/cureus.75782

**Published:** 2024-12-16

**Authors:** Dasith K Jayawickrama, Samitha Senevirathne, A H M A H Abayasinghe, Jayamini Kaushalya, Nilesh Fernandopulle

**Affiliations:** 1 Professorial Surgical Unit, National Hospital of Sri Lanka, Colombo, LKA; 2 General Surgery, National Hospital Kandy, Kandy, LKA

**Keywords:** ercp stenting, obstructive jaundice, pancreatic head masses, pancreatic sarcoidosis, systemic steroids

## Abstract

Sarcoidosis is a chronic granulomatous disease with multisystemic involvement with unspecified aetiology. Pancreatic involvement is a rare manifestation of systemic sarcoidosis and is often detected in postmortem studies. This clearly implies the rarity of the disease and its diagnostic challenges. Despite having various biochemical markers and imaging techniques, the only definitive method of diagnosis is the histological examination of the lesion. We present a patient with abdominal pain associated with obstructive jaundice, where imaging demonstrated features of pulmonary sarcoidosis along with a pancreatic head mass. Endoscopic ultrasound-guided biopsy demonstrated pancreatic non-caseating granulomas suggestive of sarcoidosis. Subsequently, the patient underwent biliary stenting and was started on glucocorticoid therapy.

## Introduction

Sarcoidosis is a multisystemic chronic granulomatous disease characterized by the presence of non-caseating granulomas without definite aetiology [1]. The annual incidence of sarcoidosis is 1-15 per 100,000 of the population without any gender predominance, with an average age at diagnosis around 50 years. It is common in northern America, Australia, and northern Europe compared to the rest of the world [2]. Although the lungs are the commonly affected organs, extrapulmonary involvement is reported in 30% of the patients with the abdomen being the most common extrapulmonary site with a frequency of 50-70% [3]. The first reported case of pancreatic involvement in systemic sarcoidosis was published by Nickerson in 1937 and found during postmortem examination [4]. Around 50% of pancreatic sarcoidosis is asymptomatic. Others may present with pain, obstructive jaundice, abdominal distention, vomiting, etc. [5]. Pancreatic adenocarcinoma, primary pancreatic lymphoma, autoimmune pancreatitis, pancreatic tuberculosis, and secondary metastatic deposits of the pancreas can be considered as differential diagnoses for pancreatic sarcoidosis. It is especially well known to mimic pancreatic and periampullary adenocarcinoma. No laboratory testing is diagnostic, and the hallmark of diagnosis is histopathological confirmation of the presence of non-caseating non-necrotizing granulomas [6]. According to available literature, endoscopic ultrasound (EUS)-guided biopsy is considered the best modality to get the tissue diagnosis of pancreatic sarcoidosis [7]. Various immune suppressive agents including glucocorticoids are proposed as the treatment for sarcoidosis with an 80% spontaneous remission rate in mild pancreatic involvement [1,8].

We present a 55-year-old male who presented with epigastric pain and was diagnosed with pancreatic sarcoidosis in the background of systemic sarcoidosis. The case is presented due to the rarity of the prevalence of pancreatic sarcoidosis. Informed consent was obtained from the patient for reporting the data.

## Case presentation

A 55-year-old previously well male presented with a two-month history of pancreatic-type epigastric pain associated with loss of appetite and loss of weight. The patient did not have a history of fever or contact with a similar patient. He occasionally drinks alcohol and doesn't smoke.

**Figure 1:**
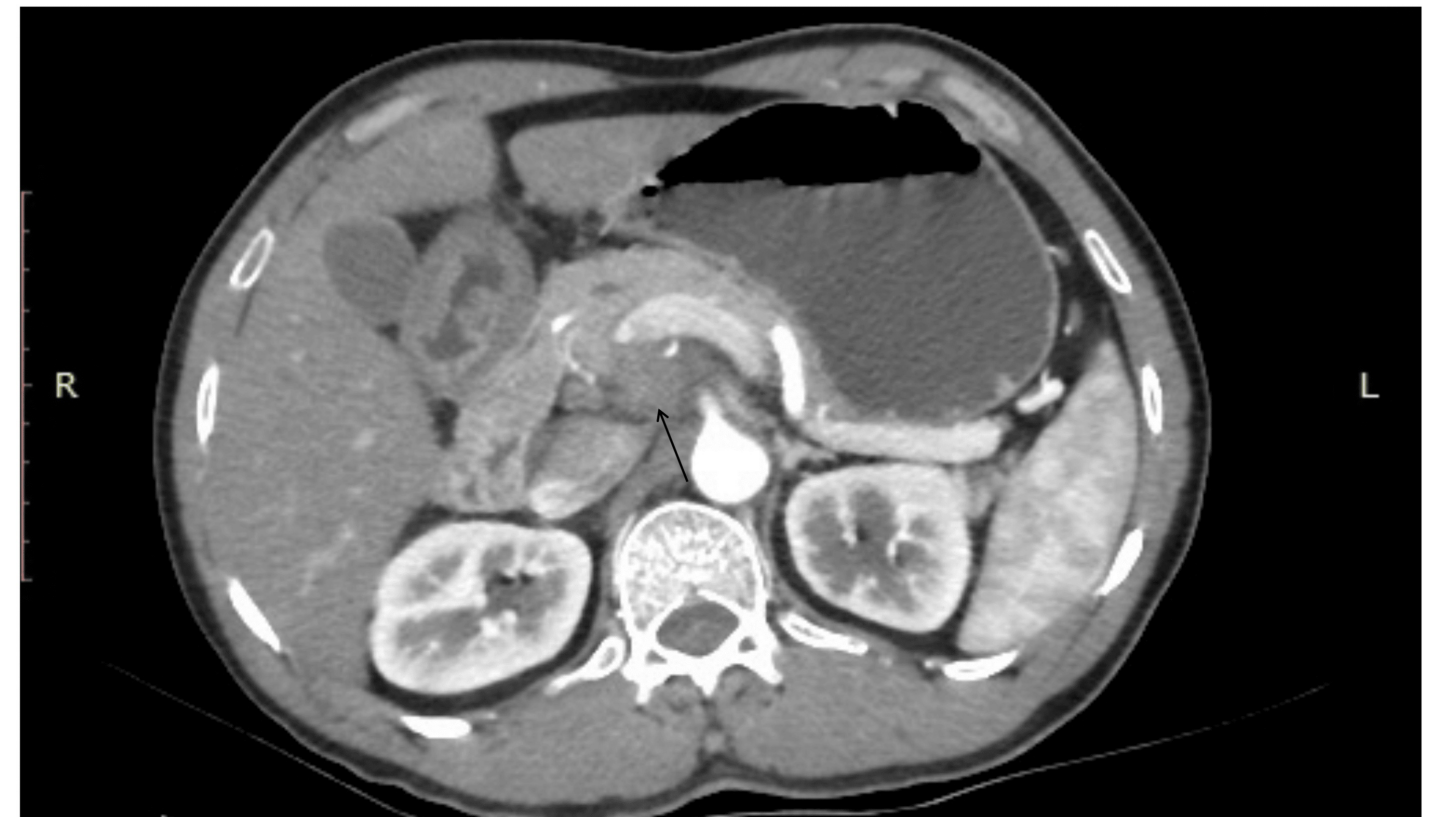
Contrast-enhanced CT of the abdomen. Ill-defined hypo-enhancing focal area in the uncinate region and enlarged celiac axis lymph nodes (arrow).

Physical examination was unremarkable. Laboratory investigations showed serum amylase of 75U/L (28-100) with normal inflammatory markers, and an ultrasound scan of the abdomen was commented as normal. Contrast CT of the abdomen revealed an ill-defined hypo-enhancing focal area in the uncinate process of the pancreas (Figure 1). The abnormal region was intimately related to the celiac axis, superior mesenteric vessels, portal vein, and left renal vein. Multiple enhancing enlarged celiac axis lymph nodes were noted. The CA 19-9 tumor marker was 20 U/ml (<35).

EUS was performed and found to have a vague hypoechoic mass in the head of the pancreas encasing the portal confluence with regional lymph nodes. Fine needle biopsy was obtained from the pancreatic lesion as well as the regional lymph nodes.

**Figure 2:**
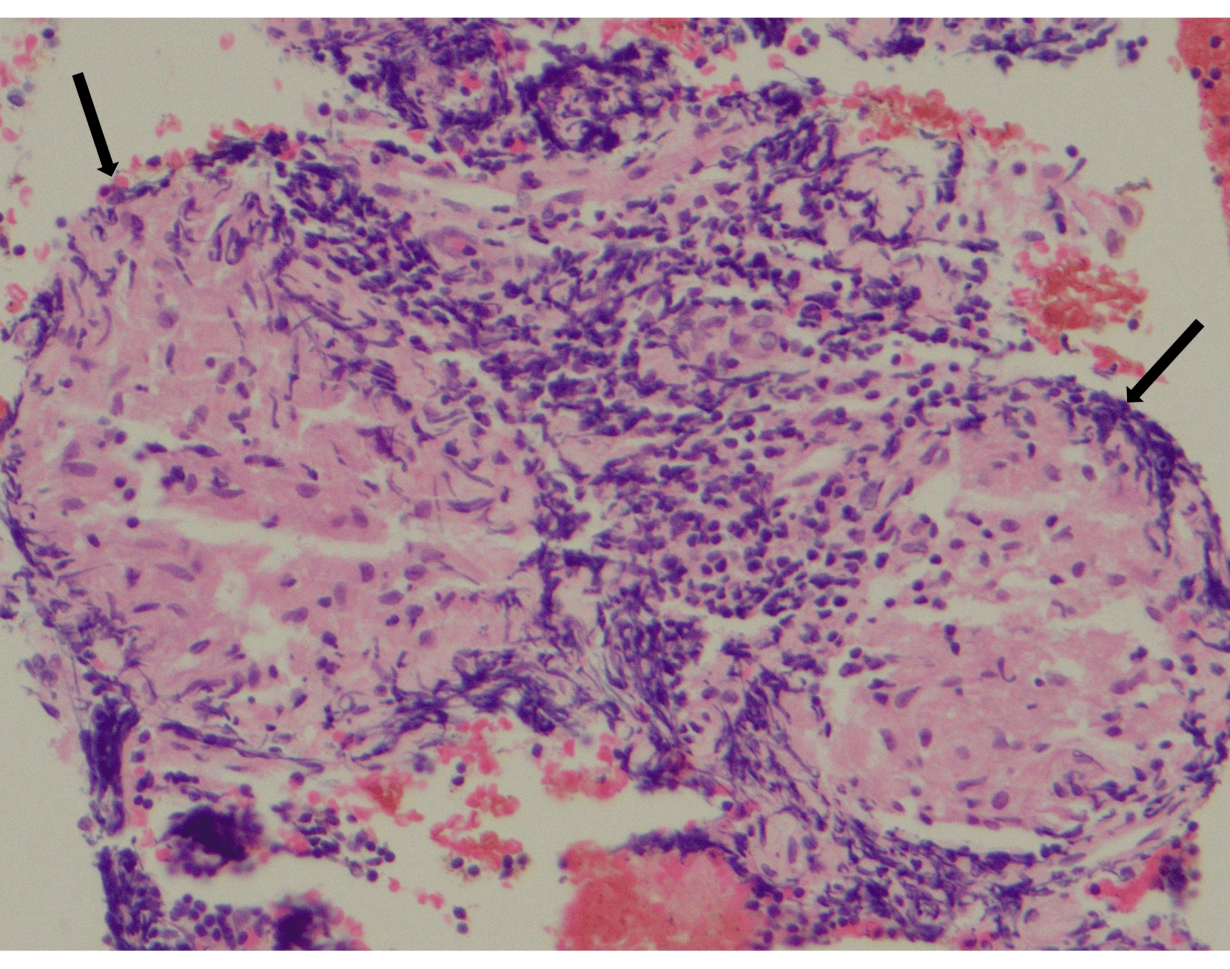
Microscopic image (H&E, ×200) of EUS-guided biopsy of pancreatic head mass. Well-formed small discrete non-necrotic epithelioid granulomata with a thin lymphoid cuff, suggestive of sarcoidosis (arrow). H&E: hematoxylin and eosin; EUS: endoscopic ultrasound

Both biopsies revealed epithelioid histiocytes forming granulomas surrounded by a cuff of lymphoid cells. No evidence of malignancy was noted (Figure 2). 

**Figure 3:**
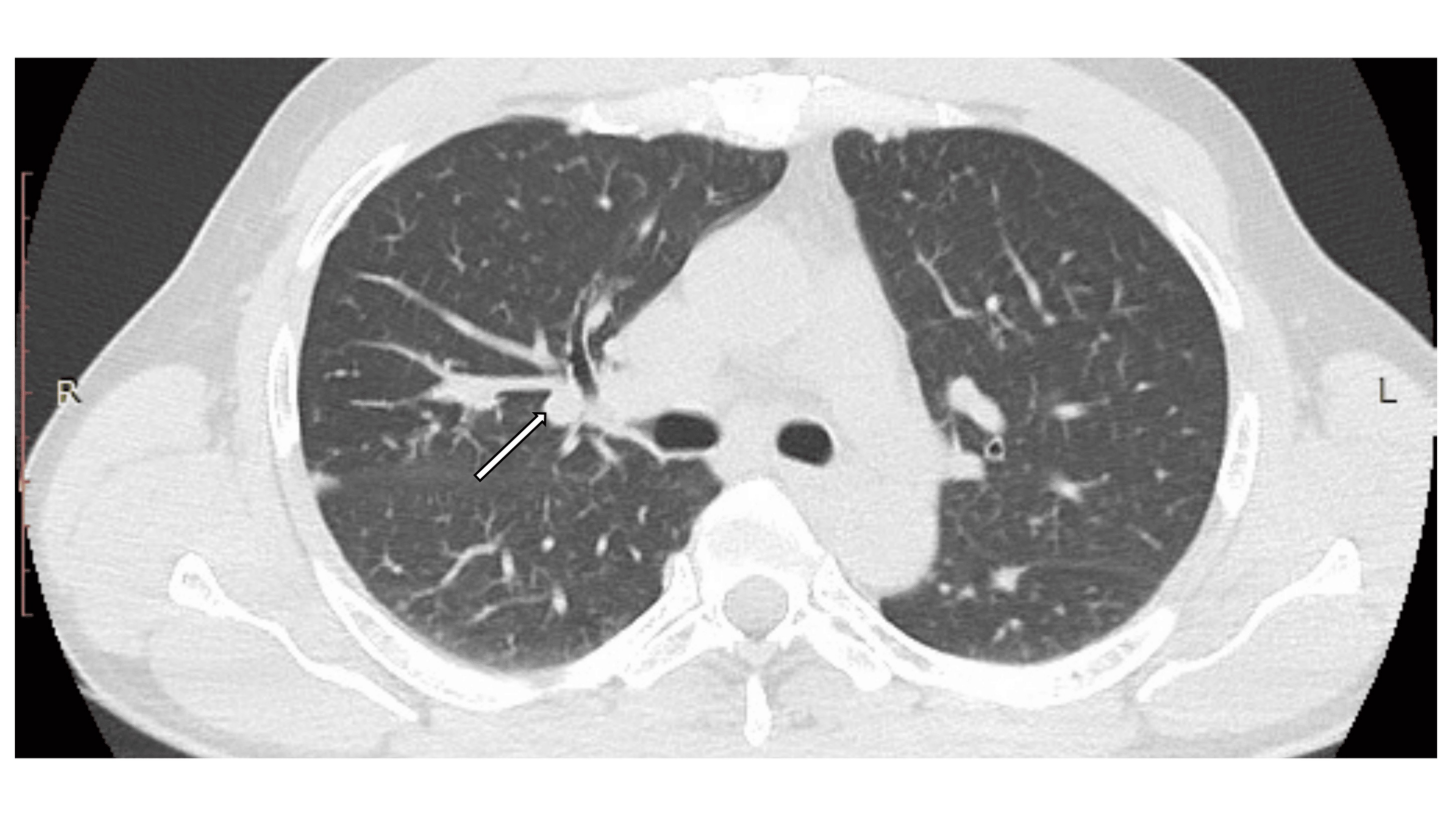
High-resolution CT of the chest. Centrilobular nodules in perilymphatic and subpleural distribution in both lungs with paratracheal lymphadenopathy, suggestive of grade 3 pulmonary sarcoidosis (arrow).

Further investigations that were carried out revealed the patient's serum total calcium level to be 9.4 mg/dl (8.5-10.2) and serum angiotensin-converting enzyme (ACE) level to be 75 U/L (8-52). Chest X-ray revealed no evidence of pulmonary sarcoidosis or tuberculosis. The Mantoux test was negative. High-resolution CT of the chest revealed centrilobular nodules in perilymphatic and subpleural distribution in both lungs with paratracheal lymphadenopathy suggestive of grade 3 pulmonary sarcoidosis (Figure 3).

During the period of investigation, the patient developed painless progressive obstructive jaundice without features of cholangitis with total bilirubin rising up to 15 mg/dl (0.2-1.2) with a direct fraction of 8.1 mg/dl. An ultrasound scan of the abdomen revealed a dilated biliary system with distal common bile duct obstruction without evidence of calculi.

**Figure 4:**
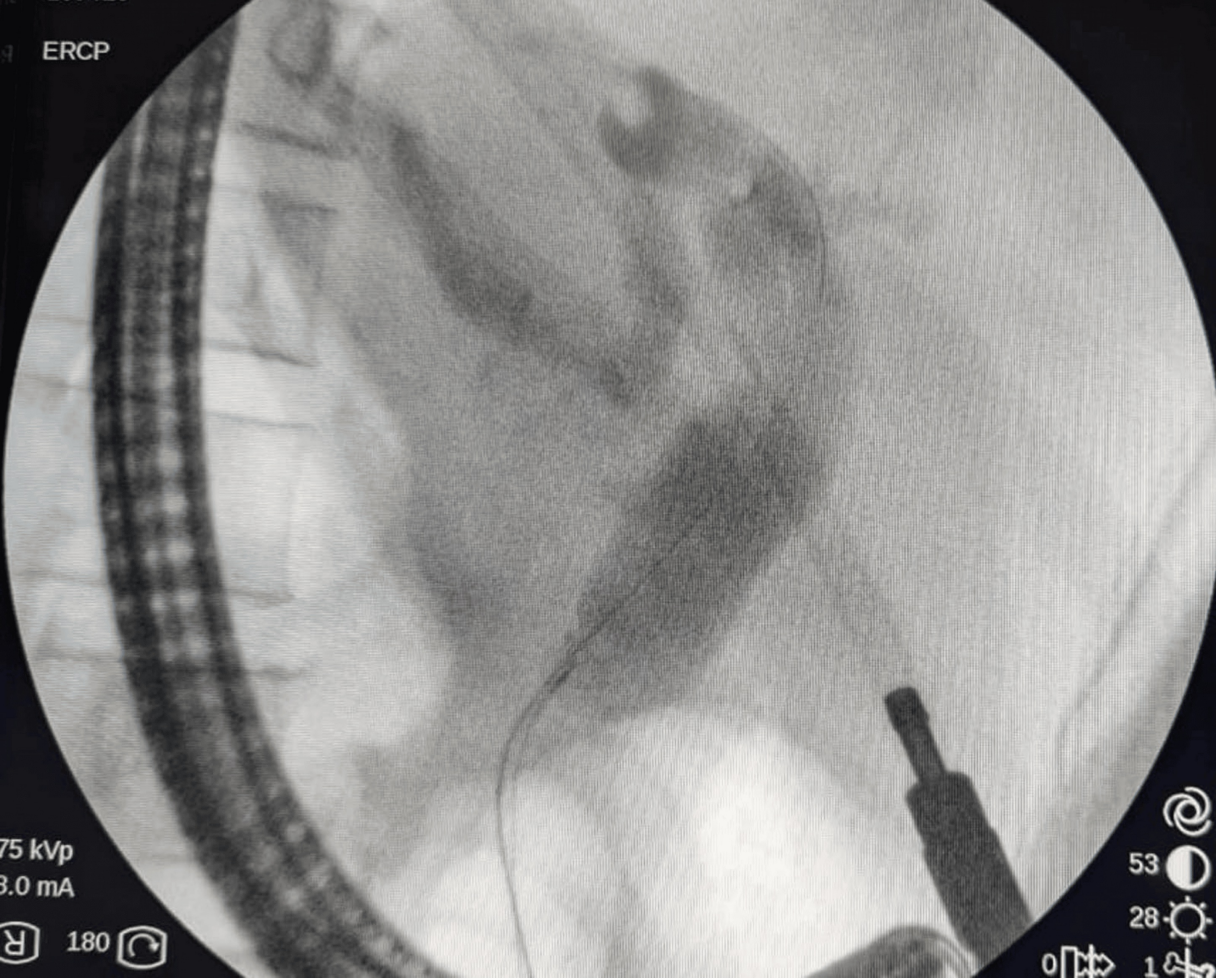
ERCP image. ERCP image showing common bile duct dilation. ERCP: endoscopic retrograde cholangiopancreatography

The patient underwent an endoscopic retrograde cholangiopancreatography to relieve the biliary obstruction, and a plastic stent was placed (Figure 4). Furthermore, the patient's condition was discussed in a multidisciplinary meeting involving a gastroenterologist, radiologist, hepato-pancreatico-biliary surgeon, respiratory physician, and rheumatologist. It was decided to start prednisolone 20 mg daily. Currently, the patient is on regular clinic follow-up with considerable improvement in pain and quality of life and awaits the interval radiological assessment of the pancreatic lesion. 

## Discussion

Pancreatic sarcoidosis is a rare manifestation of systemic sarcoidosis where only 1-5% of patients will develop. It has been detected incidentally in postmortem studies as well [9]. However, due to the low prevalence of the disease and non-specific symptoms, it is highly likely to miss a diagnosis of pancreatic sarcoidosis clinically. When it comes to imaging, no imaging modality is specific for the diagnosis of pancreatic sarcoidosis, and there are many radiological features which are common to other differential diagnoses for pancreatic sarcoidosis. Ill-defined pancreatic head mass, narrowing of the common bile duct, and enlarged lymph nodes are the commonly reported CT findings in pancreatic sarcoidosis [1]. Multiple pancreatic masses with low signal intensity on T1-weighted images, mild high signal intensity on T2-weighted images, and decreased enhancement compared to the normal pancreas after administration of gadolinium are the commonly reported MRI features [10]. However, these findings are not limited to pancreatic sarcoidosis, and other differential diagnoses should be strongly considered.

EUS-guided fine needle aspiration helps in differentiating between these conditions with similar radiological findings with a sensitivity and specificity as high as 97% [11]. The major limitation of EUS is the cost and low availability of infrastructure and expertise, especially in low-income settings. However, even though the serum ACE level is reported to be elevated in 75% of the patients with sarcoidosis, it is not considered diagnostic due to a high false positive level [12]. Although the available literature shows that the majority of pancreatic sarcoidosis are found in postmortem studies, this is a rare occasion of pancreatic sarcoidosis diagnosis following a multidisciplinary approach to a patient [13]. Therefore, the diagnosis of pancreatic sarcoidosis becomes a collective outcome of clinical, biochemical, radiological, and histopathological findings.

In our patient, considering the fact that the presence of symptoms common to the pancreatic involvement of sarcoidosis like jaundice and epigastric pain, elevated serum ACE level with a normal CA 19-9 level, CT evidence of ill-defined hypodense focal lesion in the pancreas in the background of high-resolution CT findings of pulmonary sarcoidosis, and EUS confirmation of the presence of well-formed discrete epithelioid granulomas, it was decided to manage the patient as per pancreatic involvement of systemic sarcoidosis.

## Conclusions

Pancreatic sarcoidosis which is a rare presentation can often mimic a picture of pancreatic malignancy, and definitive diagnosis is only possible with histological examination of the lesion. Since pancreatic sarcoidosis has an excellent prognosis, early identification and commencement of treatment with glucocorticoids can reduce patient morbidity.
